# Editorial: Parasites in the Tropic—A New Paradigm Shift

**DOI:** 10.3389/fimmu.2017.00509

**Published:** 2017-05-01

**Authors:** Veeranoot Nissapatorn, Yee-Ling Lau, Suleyman Yazar, Hervé Pelloux

**Affiliations:** ^1^Department of Parasitology, Faculty of Medicine, University of Malaya, Kuala Lumpur, Malaysia; ^2^Department of Medical Parasitology, Medical Faculty, Erciyes University, Kayseri, Turkey; ^3^PU-PH Parasitologie-Mycologie, Département des Agents Infectieux, Institut de Biologie et de Pathologie, CHU Grenoble Alpes, Grenoble, France

**Keywords:** parasites, the tropics, new paradigm shift

**The Editorial on the Research Topic**

**Parasites in the Tropic—A New Paradigm Shift**

The highlight of this eBook is to provide new insights into parasites from the tropics. To achieve that, much has been discussed regarding risk assessment, infection rates, disease burden, hormones and mechanism of immune response, genetic expression, and susceptibility as well as therapeutic modalities. The authors raised various hypotheses, discussed a number of concepts, and listed currently unanswered questions. The remaining important issues to resolve within the parasites from the tropics context—a new paradigm shift—are briefly covered in the following.

Since 1900, *Toxoplasma gondii* (*T. gondii*) has continuously transcended from its initial mysterious, scientific discovery to its more advanced comprehension in modern times. *T. gondii*, with felines as the definitive host, is regarded as one of the most important parasites in the tropics. Humans, as an accidental host, are the only species who still drink raw milk or milk products particularly from animal sources. The author of the first paper simplified how safe it was to drink milk in order to prevent the transmission of *T. gondii* by the insistence on heat-treating milk before consumption. It is interesting to explore how hormones play a role in *Toxoplasma* infection. Based on the systemic review from the second paper, the authors made determinations from 30 studies on humans, animals, and cell cultures. Of these, it was demonstrated that *Toxoplasma* infection was controlled by the presence of hormones found in a number of animal models. However, it is still premature to conclude which hormone has a significant relationship with *Toxoplasma* infection.

It is estimated that one-third of the world population is infected with *T. gondii* but the majority of immunocompetent persons are asymptomatic. Based on the third paper, it demonstrated that people with low prevalence of *Toxoplasma* infection do so by having close contact with animals. Also, this study leads to enhancing positive attitudes toward helping animals. For more than three decades, *T. gondii* has been considered one of the most significant opportunistic parasitic pathogens in the immunocompromised. Seroprevalence of chronic toxoplasmosis was detected in at least one-third of HIV-infected individuals in a regional hospital of southern Thailand as reported in the fourth paper. Chronic toxoplasmosis is generally an asymptomatic condition; however, 95% of cerebral toxoplasmosis (CT) cases occur as a result of secondary reactivation of chronic toxoplasmosis in AIDS patients. Thailand has successfully formulated an anti-retroviral therapy for HIV/AIDS patients and, consequently, there is a minimal incidence of AIDS-related CT in this hospital.

Clinically, CT can be a life-threatening condition and is the most common clinical disease in the immunocompromised. With this, eye involvement is the most typical extracerebral toxoplasmosis but has the best prognosis. Based on the fifth paper, the authors demonstrated that low IL-10 (Th2 response) and IFN-γ (Th1 response) concentrations were observed in OT and CT/AIDS patients. However, large amounts of TNF-α are also produced in such patients, suggesting a pronounced inflammatory response is triggered by *T. gondii*. The nature of the infecting South American strains and/or the genetic susceptibility of the host may be reasons for this type of immune response and aids in the understanding of the control mechanisms of the immune system with respect to *T. gondii*.

A great prevalence of toxoplasmosis has been reported in South America, particularly Brazil. Based on the high genetic diversity of *T. gondii* in this region, the sixth paper uniquely exhibited that *Calomys callosus* survived chronic infection by *T. gondii* clonal type II strain and was reinfected by Brazilian strains. However, congenital toxoplasmosis occurred as a consequence of the acquired immune deficiency of the host. This could be because of the reactivation of the *T. gondii* ME-49 strain and strong pro-inflammatory immune responses, including Th1 cytokines and the antibody isotype during pregnancy. Therefore, vertical transmission of *T. gondii* takes place, leading to damage of the developing fetus.

*T. gondii* is one of the TORCH agents and plays a tragic role in congenital toxoplasmosis. Yet, acute *Toxoplasma* infection in a pregnant woman and congenital complications relating to morbidity and mortality in the newborn are reported at a very low rate in Southeast Asia. Supporting this, the seventh paper conducted a questionnaire-based study on knowledge of and practice for *Toxoplasma* infection among pregnant women from three Southeast Asian nations, namely Malaysia, Philippines, and Thailand. It clearly demonstrated that health education, a core value, is the cheapest and the best option for preventive strategies to eliminate feto-maternal toxoplasmosis from this part of the world.

In terms of treatment modality, the current therapies are ineffective for congenital toxoplasmosis, chronic disease, or severe side effects that may result in serious complications. A novel experimental therapeutic synergism of diclazuril plus atovaquone combination therapy appears to elicit promising outcomes with no toxicity when treating congenital toxoplasmosis, as was demonstrated in the eighth paper. However, future trials are warranted to establish the regimen’s properties against *T. gondii* in different clinical scenarios of human toxoplasmosis in order to hone it into a more effective option. In the ninth paper, the author discussed the pathogenesis of maternal and congenital toxoplasmosis, the current treatment methodologies in clinical practice and promising experimental treatment approaches. Overall, this protozoan represents the most extraordinary example of a tropical parasite, one that sparks scientific imagination. However, there are still many challenges ahead and more exploration of *T. gondii*, the parasite that never dies, is needed.

Among parasitic diseases in Africa, one might also tend to think of a classic example of a neglected tropical disease (NTD), and human African trypanosomiasis (HAT), or sleeping sickness, comes to mind. HAT is caused by *Trypanosoma brucei rhodesiense* and *Trypanosoma brucei gambiense*; the former causes the acute form and the latter evokes the chronic type. Based on the findings from the tenth paper, it is interesting to that the common gene targets between *Glossina p. gambiensis* and *Glossina m. morsitans* have been identified and might shed light on suitable therapeutic candidates for controlling both the acute and chronic forms. Thus, continued investigation, particularly using proteomic analysis to ascertain the corresponding genes and proteins, as well as the functional roles they play, may help the search for more efficacious agents.

## Author Contributions

VN: Data collection from the published articles and writing of this manuscript. YLL, SY, and HP contribute to the writing of the manuscript with comments. All authors have read and approved the final version of the manuscript.

## Conflict of Interest Statement

The authors declare that the research was conducted in the absence of any commercial or financial relationships that could be construed as a potential conflict of interest.

